# The Choice of Calcium Salts

**Published:** 1892-08-20

**Authors:** 


### THERAPEUTICS.

### THE CHOICE OF CALCIUM SALTS.

The therapeutic value of calcium salts has long been recognised, as well as the important part they play as constituents of the human frame; but there are difficulties in the way of their absorption, so that doubts have been cast on the possibility of supplying them when they are deficient. Still, the local action of some of these as antacids and astringents is constantly made use of. The common lime water is invaluable both for external use and for the relief of certain forms of dyspepsia and vomiting. Yet it is almost inabsorbable. The greater portion passes out direct by the bowel, and only a very small part is converted by the gastric juice into a chloride, and so becomes capable of entering the blood. The same is true of the tribasic and neutral phosphates.

Recent investigations have brought out strongly the importance to many of the chief physiological functions of the presence of calcium salts. This has been shown especially with regard to the coagulation of milk in digestion, the activity of cells, the formation of certain ferments, and the power of the blood to coagulate in the mouth of wounded vessels. Are there any reliable methods of administering them? Now Germain Seé has pointed out that some of the calcium salts are far more easily absorbed and fixed in the body than the common chalk preparations, and that this is notably the case with those formed by the halogens, viz., the bromide, iodide, and chlorides which are also of remarkable efficacy in dyspepsia and stomach affections. The percentage of bromide and iodide in their calcium salts is greater than in any other of their compounds, while the calcium has neither the depressing effect of potassium nor uselessness of sodium, but is of itself valuable. In contradistinction to the inabsorbable carbonates, a considerable percentage of these salts pass out through the kidneys in some form or other. Germain Seé reports that he has had very great success in using the chloride for the treatment of stomach catarrh and for the dyspepsia of phthisis, for which he considers it invaluable. Milk especially is digested easily, and forms a friable clot in cases where it would otherwise fail to be assimilated. Again, when the digestion is unable to cope with fatty foods, the relief given by calcium chloride is very marked. Both the bromide and iodide salts act as sedatives to the stomach instead of irritating it. Though Hammond, some time ago, recommended the iodide for nerve diseases, it has not found much favour, but Seé points out that we should employ the exsiccated salts instead of the crystals, if we would wish to get a stable composition, and to know how much we are really administering. The iodide seems to possess certain antiseptic properties. The ordinary carbonates have long had a reputation for affording relief for some varieties of vesical calculus. Their value is recognized, yet it does not seem to be due to any solvent action, but probably to their astringent and sedative effect on the bladder. Spanton has found the iodide of much use in chronic cytitis; whether this is from its antiseptic or astringent effects is doubtful. In chronic Bright's disease, especially that form which follows an acute attack, the soluble lime salts increase the flow of urine and lessen the dropsy. We have recently seen the albumen diminished to a half, or even less by their use. Nearly forty years ago Rigly and others noticed the efficacy of the chloride in the hæmorrage produced by uterine fibroids. It was even hoped that a cure had been found for the disease, and Sir Spencer Wells suggested that it brought about calcification of the vessels, and thus retarded the growth of the tumour. However, the remarkable discoveries of Dr. Wright on its power in aiding coagulation of the blood indicate that it probably acts merely as a styptic. The value of lime salts, which can be really absorbed, is apparent in many other diseases. The utility of the hypophosphites has been, however, much debated, and the lacto-phosphates, which were introduced as being highly soluble, have the objection that in some of the cases where we wish to use them the tissues are already too acid. Beddoes, Fourcroy, and Begbie strongly recommended the use of calcium chloride in the treatment of strumous glands and tabes abdominalis. If we seek to imitate nature's mode of cure in incipient phthisis, we are led to seek some method of rapidly depositing lime salts around the centres of infection, and Dr. Sawyer has found in chronic phthisis the best results from the administration of calcium chloride, results better than those given by the hypophosphites. It is probable that only its long-continued use will be effectual, and the question of the dose required is important. Many patients seem capable of taking without discomfort for a time even drachm doses of the crystallized salt, but it is pointed out that large doses sometimes give rise to feelings of oppression and to diarrhœa. Rabuteau, indeed, declares that excessive ones act as a muscular poison, like potash, with a lowered temperature, slowed pulse, and arrest of the heart's action. The relation of calcium to the poisonous strontium and barium must not be overlooked here, but Brunton mentions that the lethal activity on frogs of the chloride of calcium is less than that of any other alkali or alkaline earth. The chloride and iodide have both been employed with advantage in chronic bronchitis, and Van der Corput, as Phillips notices, administered the former with cod liver oil as a jelly flavoured with anise. Finally, it has been recommended for chorea.

On the whole these halogen compounds seem to merit more extended trials. Where lime salts are needed they afford an easy means of introducing them into the organism in considerable quantities, either as an astringent or for the repair of tubercular lesions and rachitic degenerations. The chloride especially is easily absorbed without destroying any part of the gastric secretion in the process, and while its value as a gastric sedative is marked, it may be also used for its effects on the kidneys and bladder. It will be interesting to discover whether any ill effects occur if the usual rapid elimination by the kidneys is checked, and whether he iodide salt ever gives symptoms of iodism in susceptible persons.

